# Identifying childhood leukemia with an excess of hematological malignancies in first-degree relatives in Brazil

**DOI:** 10.3389/fonc.2023.1207695

**Published:** 2023-06-21

**Authors:** Daniela P. Mendes-de-Almeida, Francianne G. Andrade, Maria do Perpétuo Socorro Sampaio Carvalho, José Carlos Córdoba, Marcelo dos Santos Souza, Paulo Chagas Neto, Logan G. Spector, Maria S. Pombo-de-Oliveira

**Affiliations:** ^1^ Department of Hematology, Instituto Nacional de Infectologia Evandro Chagas, Fundação Oswaldo Cruz (FIOCRUZ), Rio de Janeiro, Brazil; ^2^ Research Center, Instituto Nacional de Câncer (INCA), Rio de Janeiro, Brazil; ^3^ Division of Epidemiology and Clinical Research, Department of Pediatrics, University of Minnesota, Minneapolis, MN, United States; ^4^ Department of Pediatric Hematology, Fundação Hospitalar de Hematologia e Hemoterapia do Amazonas (HEMOAM), Manaus, Amazonas, Brazil; ^5^ Department of Pediatric Hematology, Hospital da Criança de Brasília Jose Alencar, Brasília, Distrito Federal, Brazil; ^6^ Department of Pediatric Hematology, Centro de Tratamento Oncológico e Hematológico Infantil - Hospital Regional Rosa Pedrossian (CETOHI-HRMS), Campo Grande, Mato Grosso do Sul, Brazil

**Keywords:** familial history of hematological disorders, childhood leukemia, myeloid leukemia, lymphoblastic acute leukemia, Brazil

## Abstract

**Background:**

Familial aggregation in childhood leukemia is associated with epidemiological and genomic factors. Albeit epidemiological studies on the familial history of hematological malignancies (FHHMs) are scarce, genome-wide studies have identified inherited gene variants associated with leukemia risk. We revisited a dataset of acute lymphoblastic leukemia (ALL) and acute myeloid leukemia (AML) patients to explore the familial aggregation of malignancies among their relatives.

**Methods:**

A series of 5,878 childhood leukemia (≤21 years of age) from the EMiLI study (2000–2019) were assessed. Lack of well-documented familial history of cancer (FHC) and 670 cases associated with genetic phenotypic syndromes were excluded. Leukemia subtypes were established according to World Health Organization recommendations. Logistic regression-derived odds ratios (ORs) and 95% confidence intervals (CIs) were performed and adjusted by age as a continuous variable, where ALL was the reference group for AML and conversely. The pedigree of 18 families with excess hematological malignancy was constructed.

**Results:**

FHC was identified in 472 of 3,618 eligible cases (13%). Ninety-six of the 472 patients (20.3%) had an occurrence of FHHMs among relatives. Overall, FHC was significantly associated with AML (OR, 1.36; 95% CI, 1.01–1.82; *p* = 0.040). Regarding the first-degree relatives, the OR, 2.92 95% CI,1.57-5.42 and the adjOR, 1.16 (1.03-1.30; p0.001) were found for FHC and FHHM, respectively.

**Conclusion:**

Our findings confirmed that AML subtypes presented a significant association with hematological malignancies in first-degree relatives. Genomic studies are needed to identify germline mutations that significantly increase the risk of developing myeloid malignancies in Brazil.

## 1 Introduction

Acute leukemia (AL) is the most common type of childhood cancer and has two major subtypes, acute lymphoblastic leukemia (ALL) and acute myeloid leukemia (AML), with ALL being the most prevalent. Biological differences in cell origin are associated with specific driver genetic mutations that confer distinct pathogenesis to AL subtypes (1, 2). Hypothetically, AL results from interactions between polygenic variants, genomic instability, and environmental factors (3). The mutational contribution to clonal diversification and the relevant time windows of leukemia pathogenesis in twins with concordant leukemia subtypes have been reviewed (4).

Worldwide, a familial history of cancer (FHC) is associated with autosomal dominant or recessive syndromes, including hematological malignancies (HMs) (5–8). The occurrence of myelodysplastic syndromes (MDS) and/or AML with germline mutations has led to the World Health Organization (WHO) classification of hematopoietic neoplasms, creating a category of myeloid neoplasms with a genetic predisposition conferred by silent gene mutations (9, 10). Recently, several studies have drawn attention to the increased risk of leukemia associated with HMs and the occurrence of AL in siblings (non-twins). This evidence highlights the importance of inquiring about FHC by pediatricians during the diagnosis of AL and the investigation of genetic predisposition (11).

Although genetic predisposition has been recognized in both AL subtypes, less information about FHC in childhood ALL still contrasts with robust MDS and/or AML records (12). Therefore, we revisited the characterization of childhood leukemia over the past two decades to explore the association of FHC information between first- and second-degree relatives with either hematological or non-hematological malignancies (NHMs).

## 2 Material and methods

### 2.1 Study population with case definition and data collection

In this descriptive case-only study, the obtained clinical demography and diagnostic data were explored through secondary data analysis of the project so-called “*Estudos Multi-Institucional das Leucemias Infantis: Contribuição dos Marcadores Immuno-Moleculares na Distinção de Subtipos e Fatores de Riscos Etiopatológicos*”, acronym EMiLI. This project established a network of studies linking patient ascertainment for diagnostic biomarker identification with epidemiological data in Brazil, as described in detail elsewhere (13). Immunomolecular characterization of AL subtypes was performed using the Pediatric Oncological-Hematological Research Program, National Cancer Institute, Rio de Janeiro, Brazil. For the present assessment, the inclusion criteria were consecutive incident AL cases of biological children sent for diagnostic characterization between 2000 and 2019.

**Figure 1:**
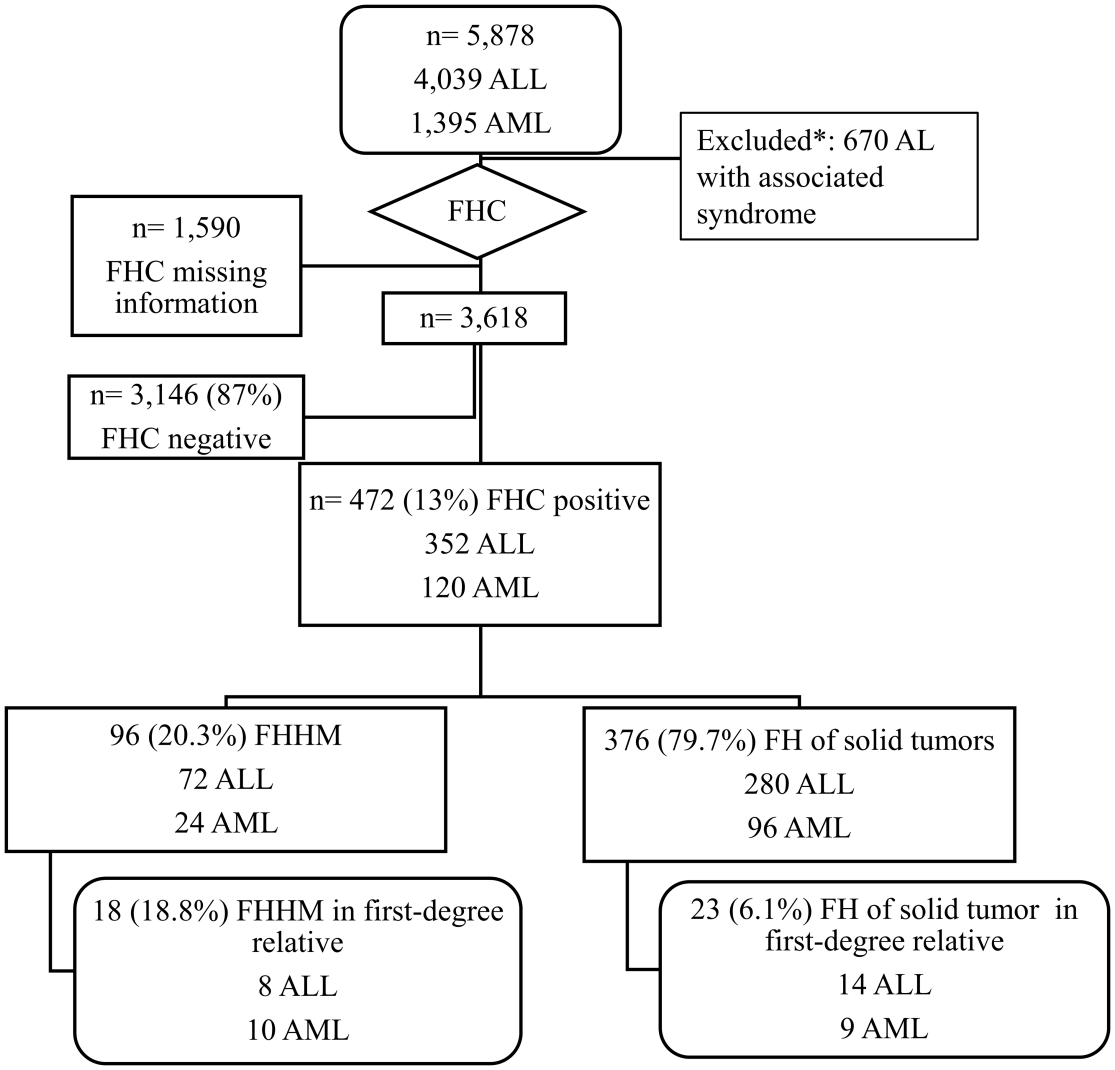
Flowchart illustrating the study design. FHC, family history of cancer; AL, acute leukemia; ALL, acute lymphoblastic leukemia; AML, acute myeloid leukemia. Down syndrome, ataxia teleangectasia, Beckwith Wiedemann syndrome, Fanconi anemia, autoimmune lymphoproliferative syndrome, Klinefelter syndrome, May-Hegglin Anomaly, neurofibromatosis, X-fragile syndrome, Wiskott-Aldrich syndrome, vitiligo, and genetic syndromes under investigation.

The childhood leukemia case definition was gathered according to the World Health Organization classification and the International Classification of Diseases for Oncology (ICD-O, 1-3) codes for HMs (14). The patients were referred for immunophenotypic and cytogenetic molecular diagnoses from medical centers in all macroregions of Brazil. The inclusion criterion was an age of up to 21 years at the time of AL diagnosis, and the exclusion criterion was the presence of an associated phenotypic genetic syndrome as summarized in the study design (**Figure 1**). For each case sent for leukemia characterization, clinical–epidemiological data were collected on ethnicity, sex, date of birth, diagnosis of AL, presence of leukemia-predisposing syndromes, place of living, and information about whether twins, first-, and/or second-degree family had cancer. The collected information on the age at diagnosis of malignancies in second-degree relatives was not detailed. The twin pairs included in this study were raised together, and consanguineous parentage was not identified. Immunophenotype karyotyping and molecular tests were performed, including karyotyping, fluorescence *in situ* hybridization (FISH), and/or PCR analysis for translocations *ETV6/RUNX1*, *TCF3/PBX1*, *KMT2A-r*, *BCR/ABL1*, and other aberrations, as well as disease status at the last follow-up. The completeness of data for each case was influenced by the period of AL occurrence and diagnostic standards. Attrition, randomization, and blinding rate were not applicable in this study.

In summary, the variables of interest were children’s sex, ethnicity, age at diagnosis (in years; date of diagnosis), period of diagnosis (in decades and data of diagnosis), type of AL (lymphoid or myeloid), medical center of treatment, type of healthcare assistance, medical and/or self-reported information on relatives with malignancies and/or hematological diseases, parenthood side of the child index, familial history of hematological malignancies (FHHMs), NHMs, and type of FHHMs (Hodgkin lymphoma, non-Hodgkin lymphoma, multiple myeloma, chronic lymphocytic leukemia, and myelodysplasia). Race/ethnicity was assessed using self-report data. Whites and non-whites were categorized into black, multiracial ethnicity, Asian, and Amerindian groups (15).

The pedigree construction of families was established in a standardized manner (16). First-degree relatives were children, parents, and siblings. Second-degree relatives were half-siblings, uncles and aunts, grandparents, grandchildren, nephews, and nieces. Third-degree relatives were cousins, great-grandparents, great-aunts, and great grand-uncles. Recollections of medical histories were performed, although adoptions, *in vitro* fertilization, and fostering children might have been underreported. The parents signed a written consent form, and the Brazilian National Institute approved this study by the Cancer Ethics and Research Committee (CEP/CONEP: # 1.394.043).

### 2.2 Statistical analysis

To compare the frequency distribution between distinct variables such as demography and childhood leukemia (ALL, AML, and hematological disorder), the χ^2^-test (two-sided) was used. The one-sample Z-test was used to compare selected cases’ proportions with well-documented FHC. The experimental variable (independent) was a family member with a history of cancer: a first-degree relative and/or second-degree relative (yes *vs.* no). The outcome variable (dependent) was ALL *vs.* AML diagnosis. A logistic regression test was used to calculate odds ratios (ORs) and performed adjusted by age as a continuous variable; 0.05 significance and 95% confidence intervals (CIs) were considered statistically significant to evaluate the association between FHC and childhood leukemia subtypes. The sample size to express the statistic’s power was calculated considering α = 0.05 and β = 0.2 for power = 0.80 (type I and II errors) and expecting the proportion of the main effect of OR of approximately 1.45 (http://powerandsamplesize.com). Analyses were performed with the IBM^®^ SPSS Statistics version 22.0.

## 3 Results

Complete information regarding FHC gathered through well-documented medical records and/or mothers’ report information was revisited in 3,618 cases (69.5%), representing the proportion of variable distribution in the selected cohort (**Supplementary Table 1**). The main demography frequency distribution of cases ascertained is shown in **Supplementary Figure 1**; in four cases, the Brazilian region origin was unknown. The ratio of white individuals to multiracial patients from the north/northeast and middle-west regions was statistically significant (*p* ≤ 0.001) in the selected group. The logistic regression-derived ORs for childhood ALL and AML with antecedents of FHC among first- and second-degree relatives of the patients are presented in **Table 1**. FHCs were documented in 472 (13.0%) patients. Overall, an increased OR was observed in patients with one relative with any type of cancer associated with AML compared to ALL (OR, 1.36; 95% CI, 1.01–1.82; *p* = 0.040). Considering only first-degree relatives with any type of cancer, the association was even higher in the AML group (OR, 2.92; 95% CI, 1.57–5.42; *p* = 0.001). Concerning parentality, the occurance of FHC in both parents showed a higher risk estimate than in the ALL group (OR, 1.62; 95% CI, 1.00–2.63), while the maternal side only was associated with ALL (OR, 1.40; 95% CI, 0.94–2.08). However, this was not statistically significant.

**Table 1:** Logistic regression-derived odds ratios (ORs) and confidence intervals (CIs) for childhood acute lymphoblastic leukemia (ALL) and myeloid leukemia (AML), with familial history of cancer in first- and second-degree relatives, Brazil, 2000–2019.

Familial history of cancer	Total	N (%)	All OR 95% CI	N (%)	AML OR 95% CI	*p*-Value
Relatives with cancer						
No	3,146 (87.0)	2,427 (87.3)	1.00*	719 (85.7)	1.00*	–
Yes	472 (13.0)	352 (12.7)	1.15 (0.92–1.44)	120 (14.3)	0.87 (0.70–1.09)	0.218
Relatives with cancer						
No antecedents	3,146	2,427	1.00*	719	1.00*	–
One relative	237 (50.2)	169 (48.1)	**0.73 (0.55–0.99)**	68 (56.2)	**1.36 (1.01–1.82)**	0.040
Two or more	190 (40.3)	145 (41.3)	0.95 (0.67–1.35)	45 (37.2)	1.05 (0.74–1.48)	0.785
Unknown**	45 (9.5)	37 (10.5)	–	8 (6.6)	–	–
First-degree relatives						
No antecedents	3,146	2,427	1.00*	719	1.00*	–
Yes	41 (8.7)	22 (6.3)	**0.34 (0.18–0.64)**	19 (15.7)	**2.92 (1.57–5.42)**	0.001
No	431 (91.3)	329 (93.7)	0.96 (0.76–1.21)	102 (84.3)	1.04 (0.82–1.32)	0.717
Second-degree relatives						
No antecedents	3,146	2,427	1.00*	719	1.00*	–
Yes	284 (60.2)	211 (60.1)	0.85 (0.65–1.13)	73 (60.3)	1.17 (0.89–1.55)	0.271
No	188 (39.8)	140 (39.9)	0.87 (0.62–1.22)	48 (39.7)	1.15 (0.82–1.61)	0.416
Parenthood side						
No antecedents	3,146	2,427	1.00*	719	1.00*	–
Maternal	177 (37.6)	146 (41.5)	1.40 (0.94–2.08)	31 (26.1)	0.72 (0.48–1.06)	0.098
Paternal	105 (22.3)	80 (22.7)	0.95 (0.60–1.50)	25 (21.0)	1.05 (0.67–1.66)	0.823
Both	77 (16.3)	52 (14.8)	0.62 (0.38–1.00)	25 (21.0)	1.62 (1.00–2.63)	0.051
Unspecified	112 (23.8)	74 (21.0)	–	38 (31.9)	–	–
Total	3,618	2,779 (76.8)		839 (23.2)		

ALL, acute lymphoblastic leukemia; AML, acute myeloid leukemia; N, number; OR, odds ratio; CI, confidence interval.

*Reference group.

**Unknown: positive family history of cancer but do not know how many.P-value means statistical significance (P < 0,05).

The 472 individuals with a positive FHC are reported in **Table 2**. For hereditary factors in malignancies, the differences between the occurrence of hematological diseases and NHMs were tested in first- and second-degree relatives with childhood leukemia. Although based on a small number of cases (n = 18), AML cases presented a higher risk estimate of having a first-degree relative with HM when compared with ALL cases, adjOR, 1.16; 95% CI, 1.03–1.30; *p* = 0.012. The logistic regression crude analysis for childhood AML in FHC was OR, 5.71; 95% CI, 1.91–17.08; *p* = 0.002, opposite to ALL with OR, 0.17; 95% CI, 0.05–0.52; *p* = 0.001 (**Supplementary Table 3**). This effect was not observed in second-degree relatives or relatives with NHMs. The most frequent HMs among the relatives were AML, B-cell precursor ALL (BCP-ALL), T-cell acute lymphoblastic leukemia, myelodysplasia, and chronic lymphocytic leukemia. The null result among first- or second-degree relatives of patients with ALL and AML with any HMs was found (**Supplementary Table 2**).

**Table 2:** Logistic regression-derived adjusted odds ratios (adjORs) and confidence intervals (CIs) for childhood acute lymphoblastic leukemia (ALL) and myeloid leukemia (AML), with familial history of cancer (FHC), Brazil, 2000–2019.

	Total N (%)	N (%)	ALL adjOR 95% CI	N (%)	AML adjOR 95% CI	*p*-Value
*Hematological malignancies*	96 (20.3)	72		24		
First-degree relatives						
No	78 (81.3)	64 (88.9)	1.00*	14 (58.3)	1.00*	–
Yes	18 (18.8)	8 (11.1)	**0.86 (0.76–0.96)**	10 (41.7)	**1.16 (1.03–1.30)**	**0.012**
Second-degree relatives						
No	52 (54.2)	37 (51.4)	1.00*	15 (62.5)	1.00*	–
Yes	44 (45.8)	35 (48.6)	1.03 (0.86–1.24)	9 (37.5)	0.96 (0.80–1.16)	0.708
*Non-hematological malignancies*	376 (79.7)	280		96		
First-degree relatives						
No	353 (93.9)	266 (95.0)	1.00*	87 (90.6)	1.00*	–
Yes	23 (6.1)	14 (5.0)	0.96 (0.88–1.05)	9 (10.0)	1.03 (0.94–1.31)	0.466
Second-degree relatives						
No	136 (36.2)	104 (37.1)	1.00*	32 (33.3)	1.00*	–
Yes	240 (63.8)	176 (62.9)	1.03 (0.95–1.11)	64 (66.7)	0.96 (0.89–1.04)	0.398

ALL, acute lymphoblastic leukemia; AML, acute myeloid leukemia; adjOR, adjusted odds ratio for age as a continuous variable; N, number; OR, odds ratio; CI, confidence interval.

*Reference group.P-value means statistical significance (P < 0,05).

**Figure 2:**
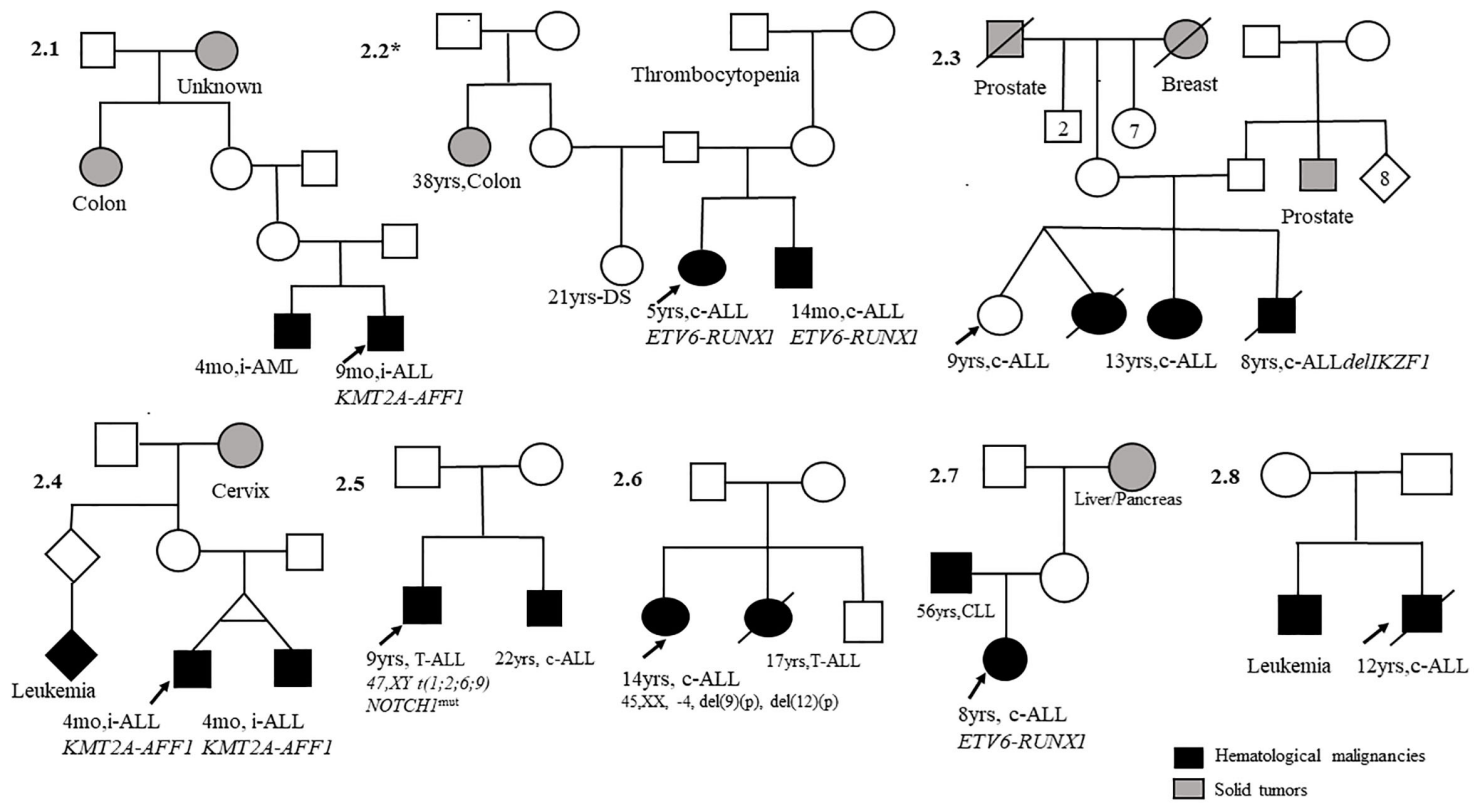
Pedigrees with child index presenting acute lymphoblastic leukemia (ALL; n = 8) first-degree relatives with hematological malignancies. mo, months; yrs, years; c-ALL, common acute lymphoblastic leukemia; T-ALL, T-cell acute lymphoblastic leukemia; CLL, chronic lymphocytic leukemia; i-ALL, infant acute lymphoblastic leukemia. DS, Down syndrome.

**Figure 3:**
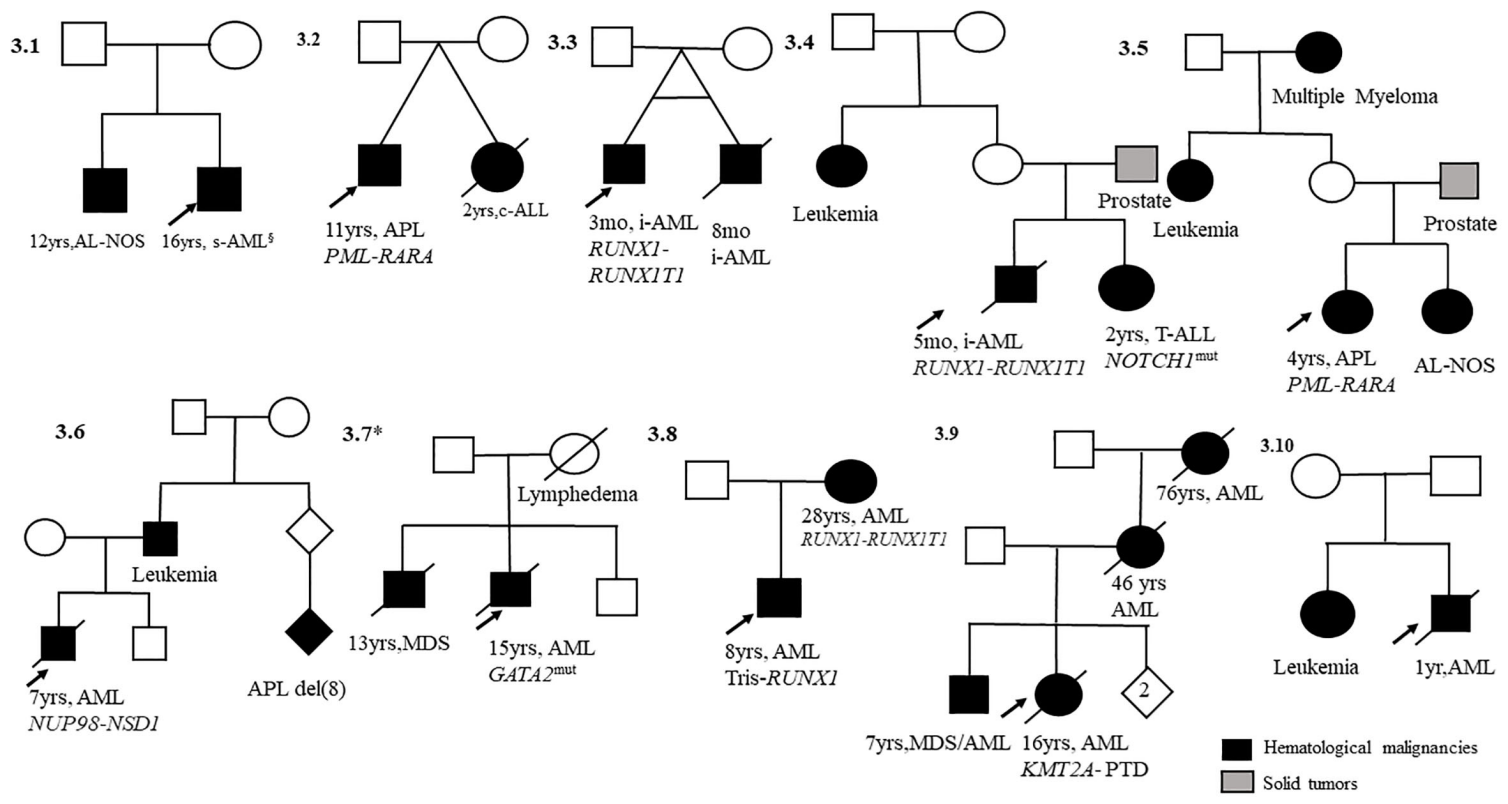
Pedigrees with child index presenting acute myeloid leukemia (AML, n = 10) and first-degree relatives with hematological malignancies. mo, months; yrs, years; AML, acute myeloid leukemia; s-AML, secondary acute myeloid leukemia after Ewing sarcoma treatment; Tris, trisomy; *GATA2* ^mut^, *GATA2* mutation; MDS, myelodysplastic syndrome; i-AML, infant acute myeloid leukemia; AL-NOS, acute leukemia not otherwise specified.

Well-documented information with diagnosis and clinical follow-up of 18 probands in eight ALL (**Figure 2**) and 10 AML (**Figure 3**) pedigrees was obtained. Somatic cytogenetic–molecular aberrations associated with distinct subtypes are also shown in **Figures 2**, **3**. There was one pair of identical infant twins with concordant ALL and *KMT2a-R* (#2.4), one pair of identical twins with AML *RUNX1-RUNX1T1* (#3.3), one identical twin set in which only one member was affected with ALL (#2.3), and one non-identical twin set with discordant leukemia subtypes, AML-M3 and c-ALL (#3.2). Additionally, there were 12 pairs of non-twin siblings and three parent–offspring duos with leukemias. The most common somatic aberrations in ALLs were *ETV6-RUNX1* (n = 3) and *KMT2a-r* (n = 3), while among AMLs, the aberrations were *RUNX-RUNXT1* (n = 3) and *PML-RARA* (n = 2). Families #2.5 and #3.7 have been reported previously (17, 18). Statistics were applied to explore the level of concordance (yes/no) of acute subtypes within the families; no significance was found (*p* = 0.61).

## 4 Discussion

This descriptive study of a nationwide cohort of Brazilian pediatric AL cases found that 13% of patients had FHC. Our findings are enriched by a well-documented familial aggregation of hematological diseases. This finding corroborates international studies, where 11% of childhood cancer survivors presented FHC or 23% of patients presented FHC (19). Although the prevalence was not assessed as population-based, the national distribution of cases in the Brazilian macroregion is like the distribution of childhood leukemia incidence, as previously described (20). An increasing trend was observed in the number of patients over time, which might be due to better access to healthcare assistance during this period and the improvement in leukemia characterization.

Overall, we observed that the odds of having one relative with cancer increased by 36% in the AML group compared with the ALL group, and almost threefold was found if the relative was a first-degree and had a hematological malignancy (OR, 2.92; *p* = 0.001). Nevertheless, after adjusting for age, we found an association risk of 10% of AMLs with FHHMs. In previous case–control studies (hospital- and population-based studies), we and others have shown an association between FHC and infant and childhood leukemia younger than 10 years at the time of diagnosis (5, 21). In Finland’s familial aggregation of early-onset lymphoma and leukemia, the risk associations were significantly elevated among first-degree relatives (22).

Factors such as inbreeding could influence FHC analysis in leukemia cases. However, this issue is rare in Brazil, and it was not fully explored herein. Although a population-based study from the United Arab Emirates found a significant twofold rate of consanguinity among ALL patients, there was no difference in the FHC between consanguineous and non-consanguineous cases (23). The excess of malignant diseases in familial occurrence might be attributable to siblings sharing common environmental exposures.

Our study found four twin sets of AL that endorse the natural history of leukemia (4). Childhood leukemia in identical twins with concordant subtypes shares the same clone-specific lesions. Hypothetically, cell aberrations originate in one twin *in utero* and spread to the second twin *via* the placental vascular pathways. The occurrence of twin pairs in the present study supports this hypothesis. This model has identified different steps in pediatric leukemia’s developmental timing, natural history, and molecular genetics (4). One pair of monozygotic twins (#2.4) who shared a unique vascular placenta developed ALL (both with the *KMT2A-AFF1* fusion gene at the same time at 4 months of age), and the second pair (#3.2), who was dizygotic, had an independent placenta and a distinct leukemia type (24). In the identical twin set with AML (#3.3), the onset disease time difference was 5 months, and the *RUNX1-RUNX1T1* aberration was only identified in one twin. Another pair of twins (#2.3) described herein were probably monozygotic twins (two girls), in which only one developed ALL. These cases suggest that placental status is critical for the risk of concordant leukemia in twins (22–24) and environmental exposure. Indeed, data from these twin sets and siblings suggest that epidemiological studies should explore exposure during the gestational period, genomic profiles, and genetic susceptibilities (4, 25, 26).

The familial aggregation of leukemia in siblings with somatic aberrations such as *ETV6-RUNX1*, *KMT2-A-r*, and *NOTCH1* mutations that we have previously reported (as case reports in siblings) in international collaboration and age at the time of leukemia diagnosis were significantly correlated with somatic mutations that initiate during fetal life (high hyperdiploidy, *ETV6-RUNX1*, *KMT2A*-r, *TCR*-rearrangements, and *NOTCH1* mutation) (17, 27–30). The sibships were of the same AL subtype with concordant markers (BCP-ALL, T-ALL, and/or AML) and shared the same cytogenetic aberrations. These data indicate a strong interaction between the genetic and environmental risk factors for childhood AL.

Large-scale high-throughput sequencing studies have recently uncovered genetic germline variants that support the premise that acute leukemia subtypes have a polygenic landscape involved in abnormal clone evolution. As increased identification of cancer predisposition syndromes has been recognized, a critical investigation of patients with HMs must also be referred for surveillance and care. In addition, the application of high-throughput sequencing technologies is essential to estimate the clinical value of low- and high-penetrance genes associated with the risk of BCP-ALL and AML. Familial clusters of leukemia led to the identification of germline variants associated with lymphoid neoplasms (*PAX5*, *IKZF1*, *SH2B3*, and *ARID2*), myeloid neoplasms (*RUNX1*, *GATA2*, *CEBPA*, *DDX41*, *ANKRD26*, *ETV6*, and *TP53*), and inherited bone marrow syndromes (*GATA2*, *TERC*, *TERT*, *FANCA*, and *FANCB*) (31). For instance, we found that *IKZF1* and *CEBPe* variants were associated with a low risk of early-age acute leukemia compared with previous international studies. In contrast, *ARID5B* rs10821936 is associated with an increased risk of AL with *MLL-MLLT3* in both ALL and AML (32). Recently, we observed an increasing number of families with multiple MDS/AL and *GATA2* mutations in germline cells (18, 33).

Study limitations must be addressed, such as the case-only design and potential bias. Case-only studies are classically used to examine the association between interactions, without involving an external control group (34), and this approach has limitations. Therefore, the case selection bias from centers collaborating on the project may have been overestimated. Another limitation is the lack of systematic information regarding the age of all relatives’ cancer diagnoses or history of smoking and/or drinking alcohol during pregnancy, as well as the exposure to ionizing radiation or pesticides, regardless if they lived in the rural zone. The validity of self-reported FHC is a critical issue, and people may interpret the data collected through face-to-face interviews or questionnaires with skepticism. However, a broad systematic review found consistent patterns across studies. It was concluded that for some cancer sites (e.g., pancreatic cancer, lung cancer, leukemia, and lymphoma), self-reported FHC could be considered sufficiently valid to be helpful in preventive counseling because of its high positive predictive value and sensitivity (>70%) (35). Another concern of the study is not to preclude selection bias, as many potential subjects were excluded for missing data, and exclusion seems to be differential with respect to ethnicity and Brazilian macroregion. In the cases of intrafamilial hematological diseases reported here, the diagnoses were confirmed by professionals who treated the children.

The strengths of our study include the national setup of a large cohort of childhood leukemia patients with detailed immunomolecular characterization that was performed in the same laboratory and the application of a well-structured questionnaire to collect additional medical information to avoid memory biases. This FHC survey provides a simple screening tool for gene–environment interactions in disease etiology.

Our findings add new data on the association of childhood AML subtype and HMs previously described in adults. Identifying children with an increased risk of hematological malignancies would be beneficial for early targeted cancer screening, genetic counseling, and surveillance programs. Further genomic studies exploring the aggregation of leukemia are crucial to elucidate the role of genetic background and ancestry as risk factors for the plausibility of the high incidence of AML in Brazil.

## Data availability statement

The original contributions presented in the study are included in the article/**Supplementary Material**, further inquiries can be directed to the corresponding author.

## Ethics statement

Brazilian National Institute approved this study by the Cancer Ethics and Research Committee (CEP/CONEP: # 1.394.043). Written informed consent to participate in this study was provided by the participants’ legal guardian/next of kin.

## Author contributions

DM-d-A: conceptualization, formal analysis, writing the original draft, review, and editing. PC: data curation and statistical analysis. FA, MSC, JC, and MSS: clinical and diagnostic investigation data and methodology. MSP-O and LS: conceptualization, funding acquisition, writing review, and editing. All authors contributed to the article and approved the submitted version.

## Supplementary material

Supplementary Figure 1 Map with the main variables analyzed according to Brazilian macroregions, 2000-2019. A total of 3,614 samples of known origin were included in the study: north/northeast, n=1,472 samples (40,7%); south/southeast, n=1,233 samples (34,1%); and midwest, n=909 samples (25,2%). Sex, ethnicity, and type of hematologic disorder were analyzed for each macroregion. BCP-ALL, B-cell precursor acute lymphoblastic leukemia; T-ALL, T-cell acute lymphoblastic leukemia; ALL NOS, acute lymphocytic leukemia not otherwise specified; AML, acute myeloid leukemia.

Supplementary Table 1 Demography and frequency distribution of hematological disorders in the selected cohort, Brazil, 2000-2019.

Supplementary Table 2 Subtypes of hematological malignancies in children, adolescents, and young adults with acute leukemia, Brazil, 2000-2019.

Supplementary Table 3 Logistic regression-derived odds ratios (ORs) and confidence intervals (CIs) for childhood acute lymphoblastic leukemia (ALL) and myeloblastic leukemia (AML), in familiar history of cancer (FHC), Brazil, 2000-2019.
