# Supplementary figures and images for: Identifying childhood leukemia with an excess of hematological malignancies in first-degree relatives in Brazil
Source: Front Oncol. 2023 Jun 21;13:1207695. doi: 10.3389/fonc.2023.1207695 (PMC10322205; doi:10.3389/fonc.2023.1207695)

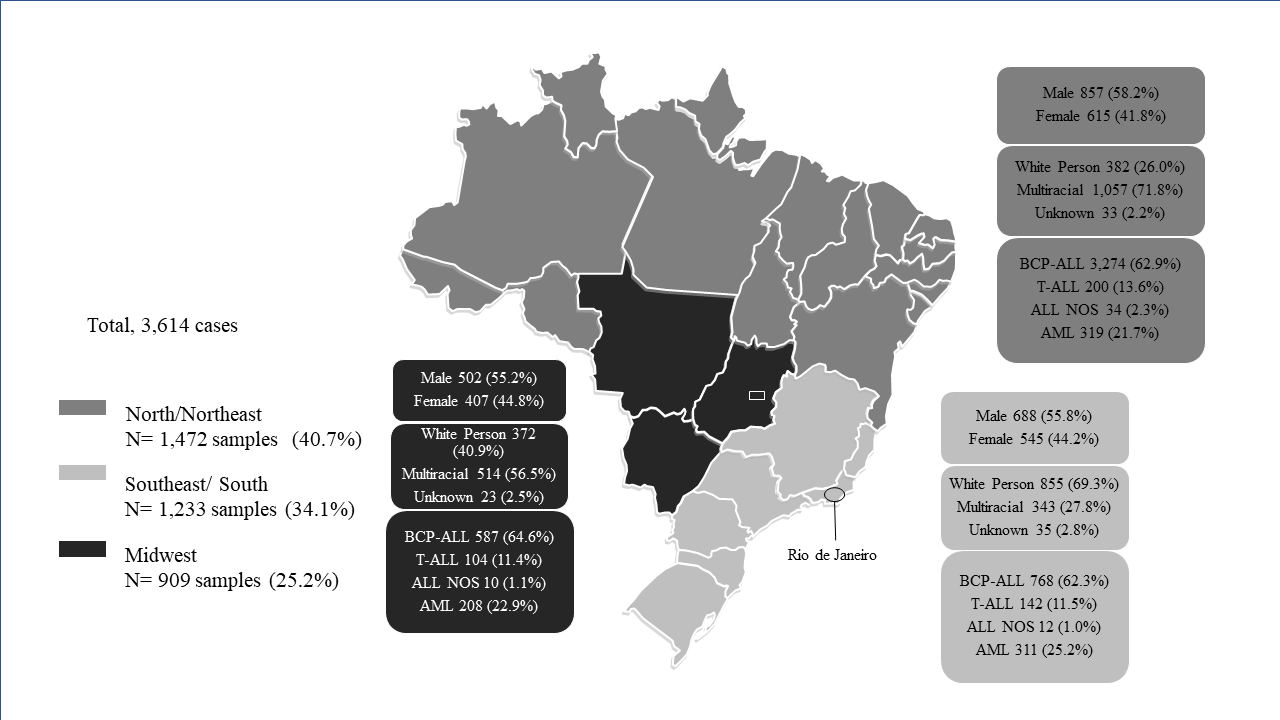

Supplement: Supplementary Figure 1 — Map with the main variables analyzed according to Brazilian macroregions, 2000-2019. A total of 3,614 samples of known origin were included in the study: north/northeast, n=1,472 samples (40,7%); south/southeast, n=1,233 samples (34,1%); and midwest, n=909 samples (25,2%). Sex, ethnicity, and type of hematologic disorder were analyzed for each macroregion. BCP-ALL, B-cell precursor acute lymphoblastic leukemia; T-ALL, T-cell acute lymphoblastic leukemia; ALL NOS, acute lymphocytic leukemia not otherwise specified; AML, acute myeloid leukemia. [file Image_1.tif]
