# Supplementary material for: Identifying childhood leukemia with an excess of hematological malignancies in first-degree relatives in Brazil
Source: Front Oncol. 2023 Jun 21;13:1207695. doi: 10.3389/fonc.2023.1207695 (PMC10322205; doi:10.3389/fonc.2023.1207695)
Supplement: Supplementary Table 1 — Demography and frequency distribution of hematological disorders in the selected cohort, Brazil, 2000-2019. [file Table_1.docx]

Supplementary Table 1. Demography and frequency distribution of hematological disorders in the selected cohort, Brazil, 2000-2019.

|  | **Total**  **N, 5208 (%)** | **Included**  **N, 3618 (%)** | **Excluded**  **N, 1590 (%)** | ***p*-value*** |
| --- | --- | --- | --- | --- |
| Leukemia subtypes |  |  |  |  |
| BCP-ALL | 3,196 (61.4) | 2,277 (62.9) | 919 (57.8) | 0.06 |
| T-ALL | 666 (12.8) | 446 (12.3) | 220 (13.8) | 0.41 |
| AML | 1,236 (23.7) | 839 (23.2) | 397 (25.0) | 0.48 |
| Others | 110 (2.1) | 56 (1.5) | 54 (3.4) | 0.02 |
| Sex |  |  |  |  |
| Male | 2,2978 (57.2) | 2049(56.6) | 929 (58.4) | 0.67 |
| Female | 2,221 (42.6) | 1569 (43.4) | 652 (41.0) |  |
| Unknown | 9 (0.2) | -- |  | -- |
| Ethnicity |  |  |  |  |
| White | 2,144 (41.2) | 1,612 (44.6) | 532 (33.5) | 0.001 |
| Non-White | 2,796 (53.7) | 1,915 (52.9) | 881 (55.4) | 0.53 |
| Unknown | 268 (5.1) |  |  | -- |
| Age group (years) |  |  |  |  |
| ≤1 | 911 (17.5) | 673 (18.6) | 238 (15.0) | 0.09 |
| 2-5 | 1.754 (33.7) | 1,232 (34.1) | 522 (32.8) | 0.67 |
| 6-10 | 1,185 (22.8) | 812 (22.4) | 373 (23.5) | 0.62 |
| 11-21 | 1,358 (26.1) | 901 (24.9) | 457 (28.7) | 0.11 |
| Brazilian macroregions |  |  |  |  |
| North/Northeast | 2,221 (42.6) | 909 (25.1) | 225 (14.2) | 0.001 |
| Midwest | 1,134 (21.8) | 1,472 (40.7) | 749 (47.1) | 0.001 |
| South/Southeast | 1,797 (34.5) | 1,233 (34.1) | 564 (35.5) | 0.61 |
| Unknown | 58 (1.1) | 4 (0.1) | 52 (3.3) | -- |
| Total | 5,208 (100.0) | 3,618 (69.5) | 1,590 (30.5) |  |

^*^*p*-value calculated by Z-test; BCP-ALL- B-cell acute lymphoblastic leukemia; T-ALL- T-cell acute lymphoblastic leukemia, AML- Acute myeloblastic leukemia, Brazil, 2000-2019.
