# Supplementary material for: Identifying childhood leukemia with an excess of hematological malignancies in first-degree relatives in Brazil
Source: Front Oncol. 2023 Jun 21;13:1207695. doi: 10.3389/fonc.2023.1207695 (PMC10322205; doi:10.3389/fonc.2023.1207695)
Supplement: Supplementary Table 2 — Subtypes of hematological malignancies in children, adolescents, and young adults with acute leukemia, Brazil, 2000-2019. [file Table_2.docx]

Supplementary Table 2. Subtypes of hematological malignancies of index- children, adolescents, and young adults with acute leukemia and relatives, Brazil, 2000-2019

| **Hematological malignancies subtypes**  **among relatives** | **Total** | **ALL**  **N (%)** | **ALL**  **Crude OR**  **(C.I. 95%)** | **ALL**  **Adj OR***  **(C.I. 95%)** | **AML**  **N (%)** | **AML**  **Crude OR**  **(C.I. 95%)** | **P-value** | **AML**  **Adj OR***  **(C.I. 95%)** | **P-value** |
| --- | --- | --- | --- | --- | --- | --- | --- | --- | --- |
| No antecedents** | 3,146 | 2,427 | 1.00* | 1.00* | 719 | 1.00* | - | 1.00* | - |
| Leukemia and MDS | 50 (86.2) | 36 (87.8) | 0.76 (0.41-1.42) | 0.65 (0.35-1.22) | 14 (82.4) | 1.31 (0.70-2.45) | 0.392 | 1.54 (0.82-2.89) | 0.181 |
| Lymphoma, Multiple Myeloma | 8 (13.8) | 5 (12.2) | 0.49 (0.12-2.07) | 0.44 (0.10-1.86) | 3 (17.6) | 2.02 (0.48-8.49) | 0.335 | 2.29 (0.54-9.79) | 0.263 |
| Total | 58 | 41 (70.7) |  |  | 17 (29.3) |  |  |  |  |

Abbreviations: ALL- Acute lymphoblastic leukemia*;* AML- Acute myeloid leukemia; OR- Odds ratio. MDS- Myelodysplastic syndromes. *Adjusted by age and ethnicity ** Subjects without family history of cancer.
