# Supplementary material for: Identifying childhood leukemia with an excess of hematological malignancies in first-degree relatives in Brazil
Source: Front Oncol. 2023 Jun 21;13:1207695. doi: 10.3389/fonc.2023.1207695 (PMC10322205; doi:10.3389/fonc.2023.1207695)
Supplement: Supplementary Table 3 — Logistic regression-derived odds ratios (ORs) and confidence intervals (CIs) for childhood acute lymphoblastic leukemia (ALL) and myeloblastic leukemia (AML), in familiar history of cancer (FHC), Brazil, 2000-2019. [file Table_3.docx]

Supplementary Table 3**.** Logistic regression-derived crude odds ratios (ORs) and confidence intervals (CIs) for childhood acute lymphoblastic leukemia (ALL) and myeloblastic leukemia (AML), in familiar history of cancer (FHC), Brazil, 2000-2019

|  | **Total**  **N (%)** | **N (%)** | **ALL**  **OR 95%**  **CI** | **N (%)** | **AML**  **OR 95%**  **CI** | ***P*-value** |
| --- | --- | --- | --- | --- | --- | --- |
| *Hematological malignancies* | 96 (20.3) | 72 |  | 24 |  |  |
| First degree relatives |  |  |  |  |  |  |
| No | 78 (81.3) | 64 (88.9) | 1.00* | 14 (58.3) | 1.00* | - |
| Yes | 18 (18.8) | 8 (11.1) | 0.17 (0.05-0.52) | 10 (41.7) | 5.71 (1.91-17.08) | 0.002 |
| Second degree relatives |  |  |  |  |  |  |
| No | 52 (54.2) | 37 (51.4) | 1.00* | 15 (62.5) | 1.00* | - |
| Yes | 44 (45.8) | 35 (48.6) | 1.58 (0.61-4.06) | 9 (37.5) | 0.63 (0.25-1.63) | 0.519 |
| *Non-hematological malignancies* | 376 (79.7) | 280 |  | 96 |  |  |
| First degree relatives |  |  |  |  |  |  |
| No | 353 (93.9) | 266 (95.0) | 1.00* | 87 (90.6) | 1.00* | - |
| Yes | 23 (6.1) | 14 (5.0) | 0.51 (0.21-1.22) | 9 (10.0) | 1.97 (0.82-4.70) | 0.129 |
| Second degree relatives |  |  |  |  |  |  |
| No | 136 (36.2) | 104 (37.1) | 1.00* | 32 (33.3) | 1.00* | - |
| Yes | 240 (63.8) | 176 (62.9) | 0.85 (0.52-1.38) | 64 (66.7) | 1.18 (0.72-1.93) | 0.503 |

*Reference group. Abbreviations: N-number of cases; ALL acute lymphoblastic leukemia; AML- acute myeloblastic leukemia; OR-crude odds ratio; CI- confidence intervals.
